# Physiotherapist-led intermittent noninvasive ventilation for hypoxaemia following abdominal surgery in a quaternary Australian hospital: a randomised pilot feasibility trial

**DOI:** 10.1016/j.bjao.2026.100571

**Published:** 2026-07-13

**Authors:** Claire Hackett, Linda Denehy, Peter Kruger, Nina Ripley, Sharyn Furze, Natasha Reid, B. Mark Smithers, Ianthe Boden

**Affiliations:** 1 Princess Alexandra Hospital, Brisbane, Australia; 2 The University of Melbourne, Melbourne, Australia; 3 Peter MacCallum Cancer Centre, Melbourne, Australia; 4 The University of Queensland, Brisbane, Australia; 5 University of Tasmania, Launceston, Australia; 6 Launceston General Hospital, Launceston, Australia

**Keywords:** abdominal surgery, hypoxia, noninvasive ventilation, physical therapy modalities, physiotherapy, postoperative care, postoperative pulmonary complications

## Abstract

**Background:**

Physiotherapy management of postoperative hypoxaemia is understudied. This trial aimed to assess the feasibility of a trial of physiotherapist-led noninvasive ventilation (NIV) in adults with hypoxaemia following major abdominal surgery.

**Methods:**

This prospective, single-centre, parallel-group, assessor-blinded, feasibility pilot trial with concealed allocation and intention-to-treat analysis randomised adults with hypoxaemia (oxyhaemoglobin saturations <90% on room air) within 72 h of major abdominal surgery to either usual care physiotherapy (education, early mobilisation, and deep breathing and coughing exercises), or usual care plus four 30-min sessions of physiotherapist-led NIV. The primary outcome was feasibility, including patient recruitment rates, NIV treatment adherence, and acceptability to patients. Exploratory outcomes included postoperative pulmonary complications (PPCs).

**Results:**

From 50 participants, 49 were analysed. Mean age (standard deviation) was 62 (10) yr and 51% were male. Over 30 weeks, the recruitment rate was 1.7 participants per week (95% confidence interval [CI], 0.7–3.4). Mean total NIV delivered was 78 min (95% CI, 55–100) of the protocolised 120 min. There were no serious adverse events. NIV was acceptable to 91% of participants (20/22; 95% CI, 72%–97%). The PPC incidence on trial enrolment ranged from 6% (3/49; 95% CI, 2%–17%) to 47% (23/49; 95% CI, 34%–61%) depending on the measurement tool used.

**Conclusions:**

Recruitment to this trial of physiotherapist-led NIV in patients with postoperative hypoxaemia was feasible. NIV was rated acceptable by most participants, and NIV adherence similar to that of previous trials. This study provides valuable insights for the design and conduct of future physiotherapy postoperative treatment studies.

**Clinical trial registration:**

ACTRN12622000839707. https://www.anzctr.org.au/Trial/Registration/TrialReview.aspx?id=382199&isReview=tru

**Editor's key points**

• Hypoxaemia is a common complication following major abdominal surgery and is associated with increased postoperative morbidity and prolonged hospital stay.
• This randomised pilot feasibility trial demonstrated that a physiotherapist-led protocol for intermittent non-invasive ventilation (NIV) is feasible and safe to implement on a general surgical ward.
• The study successfully achieved its primary recruitment target and demonstrated high adherence rates to the intermittent NIV protocol among surgical participants.
• Preliminary clinical signals suggest that intermittent NIV may improve oxygenation and reduce the incidence of postoperative pulmonary complications compared to standard oxygen therapy.
• These findings provide the necessary feasibility data to support the design and implementation of a larger, definitively powered multicentre trial to evaluate the clinical efficacy of ward-based, physiotherapist-led NIV.

Hypoxaemia occurs in up to 65% of patients following major abdominal surgery.^1^, ^2^, ^3^ It is a serious complication associated with ICU admissions, delayed hospital discharge and increased risk of mortality.^3^, ^4^, ^5^, ^6^ Recognition of postoperative hypoxaemia may be masked or missed if routine continuous oxygen therapy remains in place during assessment.^7^ Temporary purposeful removal of postoperative oxygen therapy to detect low peripheral oxyhaemoglobin saturation (SpO_2_) levels, an Air-Test, identifies atelectasis^8^ and can predict postoperative pulmonary complications (PPCs).^9^ Various desaturation thresholds have been reported to diagnose hypoxaemia with an Air-Test.^8^, ^9^, ^10^ One example, the Air-Test-90, diagnoses hypoxaemia when SpO_2_ is lower than 90% within 2 min of breathing air.^10^ Earlier recognition of postoperative hypoxaemia may identify patients to target with treatments aimed at reversing atelectasis and minimising more serious sequelae. However, a positive Air-Test-90 is understudied as a trigger for additional therapies, including physiotherapy.

Physiotherapists routinely provide education, early mobilisation, and breathing exercises to patients following major abdominal surgery.^10^^,^^11^ Research in this field has predominantly focused on physiotherapy interventions as a primary prevention of PPC.^11^ Few studies have specifically tested physiotherapy modalities to treat postoperative hypoxaemia. Continuous therapeutic noninvasive ventilation (NIV) is recommended to treat hypoxaemia following abdominal surgery as it can improve oxygenation, reduce atelectasis, avoid reintubation and reduce mortality.^12^^,^^13^ However, access to NIV may be limited on surgical wards and post-anaesthetic care units due to organisational restrictions such as monitoring requirements and staff expertise.^14^^,^^15^

Integrating the delivery of intermittent NIV within usual care physiotherapy services may be a method of improving access to therapeutic NIV for patients with hypoxaemia. While the safety and feasibility of physiotherapist-led intermittent NIV has been previously demonstrated in high-risk non-hypoxaemic surgical patients,^16^ it is yet to be determined if the application of physiotherapy-led NIV is feasible and safe within more acute patients who have developed postoperative hypoxaemia detected with the Air-Test-90.

The primary aim of the physiotherapist-led intermittent NIV for hypoxaemia following abdominal surgery trial was to assess the feasibility of trial conduct, including the recruitment rate of patients who develop hypoxaemia within 72 h of abdominal surgery into a clinical trial, and to assess NIV adherence and patient acceptability. Secondary aims were to assess the safety of trial interventions, participant enrolment milestones, adherence to early mobilisation and respiratory interventions, and to assess clinical outcomes.^17^

## Methods

### Design and setting

This is a pragmatic, assessor-blinded pilot feasibility randomised trial undertaken at an Australian metropolitan quaternary trauma hospital between January and August 2023.

Ethical approval was provided by Metro South Human Research and Ethics Committee on 15 June 2022 (HREC/ 2021/QMS/77244). The trial was prospectively registered (ACTRN12622000839707) on 8 April 2022, and conducted and reported in accordance with the Consolidated Standards of Reporting Randomised Controlled Trials (CONSORT 2010) extensions for pilot and feasibility randomised trials,^18^ and the CONSORT extensions for non-pharmacological^19^ and pragmatic trials^20^ (Supplementary Table 1). Written informed consent was obtained for all participants. Full details of trial rationale, design, methods, protocol, and interventions are published elsewhere.^17^

### Participants

Eligible patients were adults (≥18 yr) with hypoxaemia who had abdominal surgery with either an open incision (≥5 cm) or anaesthetic time ≥3 h, extubated within 24 h of surgery, and breathing without NIV. A detailed description of eligibility criteria is in Supplementary Table 2. The research team (CH and NRi) screened the prior day’s theatre list daily for patients having major abdominal surgery, then confirmed initial eligibility with medical chart review. Patients who met all inclusion criteria and none of the exclusion criteria were screened by the investigators daily for hypoxaemia using the Air-Test-90.^17^ The Air-Test-90 diagnoses hypoxaemia if oxyhaemoglobin saturation of <90% occurs anytime during 2 min of supplemental oxygen therapy removal. For eligible patients diagnosed with hypoxaemia, investigators (CH or NRi) sought first approval for trial participation from the admitting surgical team, followed by consent from the patient or substitute decision maker. If the substitute decision maker was unable to be contacted, enrolment prior to consent was approved in line with previous trials^10^ and trial governance guidlines.^21^

### Randomisation

The randomisation schedule was generated within REDCap (Research Electronic Data Capture)^22^ by an independent administrator with no further involvement in the trial. Consenting participants were randomly assigned (1:1) via concealed allocation by investigators (CH or NRi) to receive usual care physiotherapy or usual care and physiotherapy-led sessions of intermittent NIV.

### Treatments

Participants were scheduled to receive the trial treatments from the day of enrolment until the seventh postoperative day, unless any of the following occurred first: a protocolised threshold for discharge from physiotherapy services was reached, or escalation to continuous (≥1 h) NIV, CPAP, or invasive mechanical ventilation was ordered by the treating medical team.^17^

Physiotherapists delivering trial treatments were clinicians trained in all procedures and were working on the surgical wards or ICU. The physiotherapist expertise level and the number of sessions delivered to each group were recorded.

#### Usual care physiotherapy (control)

Participants randomised to the control were provided with a bundle of three physiotherapy treatments; ‘Talk’ + ‘Walk’ + ‘Breathe’. These are commonly provided by physiotherapists to high-risk patients after abdominal surgery.^10^ ‘Talk’ consisted of at least one verbal education session on the prevention of postoperative complications with early mobilisation and breathing exercises, consolidated with written materials. ‘Walk’ was mobilisation away from the bedside for 10 to 15 min provided once daily. If unable to participate in upright walking, non-walking physical rehabilitation exercises were provided.^10^ ‘Breathe’ was supervised deep breathing (two sets of 10 breaths) and coughing exercises performed in upright sitting for a minimum of four treatment sessions over 2 days.

#### Additional physiotherapist-led noninvasive ventilation (intervention)

Participants randomised to the intervention group received additional physiotherapy-led NIV sessions to the ‘Talk’ + ‘Walk’ + ‘Breathe’ bundle. This consisted of education and orientation to NIV and four 30-min sessions delivered over two consecutive days. Bilevel NIV was delivered by a trained physiotherapist using a ResMed VPAP S9™ machine and a ResMed AcuCare F 1-1 non-vented face mask™ (ResMed, Oxfordshire, UK). Expiratory positive airway pressure was initiated at 5 cmH_2_0 then progressed to 10 cmH_2_0 and inspiratory positive airway pressure from 10 to 15 cmH_2_0 depending on patient tolerance. In line with pragmatic flexibility, additional sessions of ‘Talk’ or ‘Breathe’ for both groups, and NIV for those in the intervention group, were delivered at the treating physiotherapist’s discretion and reasons recorded.

In both groups, high flow oxygen therapy (HFOT) was not protocolised. If prescribed by the medical team in line with local practice, this was recorded. Surgical, anaesthetic, and nursing practice were not protocolised and were conducted according to local clinical practices.

### Outcomes and follow-up

The primary outcome was the feasibility of trial conduct, assessed by first, recruitment rate—number of eligible patients recruited per week; second, adherence to NIV protocol—mean cumulative total NIV delivered (min) for intervention participants; third, acceptability of treatments to participants and clinicians assessed with a customised acceptability questionnaire based on the theoretical framework of acceptability.^17^

#### Safety

We measured the number of adverse events per treatment and group, occurring during or within 15 min of a treatment session.

- Serious adverse event: Any event attributable to trial treatments that results in death, is life-threatening, requires prolongation of hospitalisation, or results in persistent or significant disability or incapacity.

- Adverse event: An unintended deterioration in medical condition attributable to trial treatments that does not resolve when treatment has ceased, requiring medical team review and a change in medical management.

- Transient physiological events: A temporary physiological change (vital signs) or patient-reported condition that resolves with cessation of treatment.

Harms were monitored with planned assessment of the patient’s vital signs by the treating physiotherapist during and 15 min following therapy sessions or if recorded by other clinicians in the medical record.

Additional secondary outcomes included further measures of the recruitment process, the feasibility of delivering trial protocols, and adherence to treatments. Exploratory efficacy outcomes included onset of a PPC within seven postoperative days, escalation of acute care, change in health-related quality of life (EuroQol [EQ5D-5L])^23^ from preadmission to 90 postoperative days, and at 30 and 90 postoperative days; hospital re/admission, days alive and out of hospital, and mortality. Details of data acquisition and outcomes are described elsewhere.^17^ Changes made during the trial to planned methods are reported in Supplementary Table 3.

The performance of feasibility domains in this pilot trial was designed to contribute equally towards determining this trial’s feasibility, whether progress to future study was supported, and to build recommendations for further trial design and conduct. Feasibility thresholds and progression criteria were not predefined.

#### Blinding

Assessors and the trial statistician were blinded to group assignment. Attempts to minimise ward staff awareness of group allocation were taken, such as drawing curtains, removing NIV from the bedside between treatment sessions, and medical record documentation of NIV delivery was separated, then later reintegrated at completion of the participant’s episode of care. Treating physiotherapists and participants could not be blinded due to the nature of this study. The success of assessor blinding was not evaluated.

#### Statistical methods

The sample to assess trial feasibility was based on anticipated non-recruitment of 20% of eligible patients due to exclusion criteria.^17^ Details of data handling were described previously.^17^ Missing data were not imputed. Analyses were conducted on available cases, which were exploratory and unadjusted. Analyses were prespecified; no post hoc analyses were conducted.

Descriptive statistics expressed the characteristics of each treatment group. Continuous data were summarised by mean and standard deviation (sd) if normally distributed, and median and interquartile range (IQR) if non-normally distributed. Categorical data were summarised using frequencies and proportions. Treatment groups were compared at baseline using Student’s *t*-tests, χ^2^ tests, and their non-parametric equivalents as necessary. Primary trial feasibility and protocol adherence outcomes were summarised descriptively. Secondary feasibility outcomes of recruitment processes, therapy delivery fidelity, and response to therapy were compared between groups using odds ratios for binary outcomes, Student’s *t*-tests for continuous variables, χ^2^ tests for categorical variables, and appropriate non-parametric equivalents for data that were not normally distributed. Secondary explorative outcomes were assessed using repeated measures or time-to-event analyses, depending on the outcome. Primary models were unadjusted and analysed according to intention-to-treat principles unless otherwise stated. *P*-values are two-sided with <0.05 considered statistically significant. Statistical analysis was conducted by a biostatistician blinded to treatment allocation.

## Results

### Trial processes

**Fig 1:**
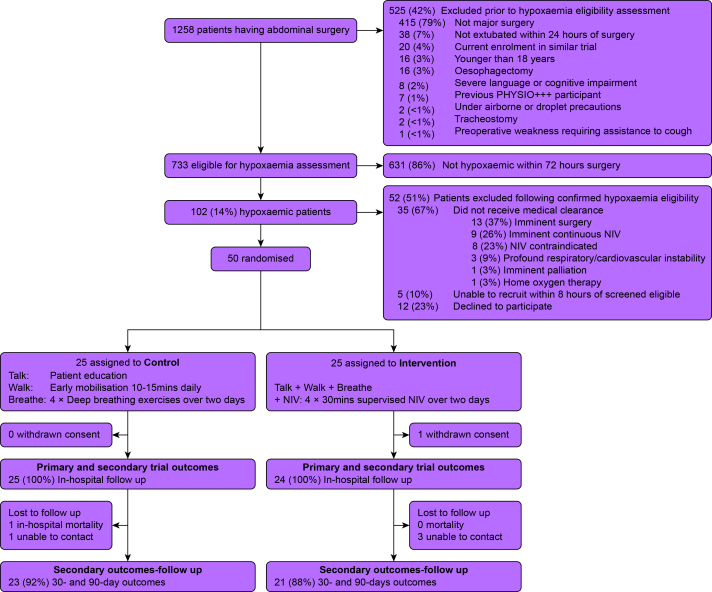
Flow of PHYSIO+++ participants through the trial. NIV, noninvasive ventilation. Proportions may not add up to 100% due to rounding.

Between January and August 2023, of 1258 patients having abdominal surgery, 733 patients were potentially eligible and screened for hypoxaemia (Fig. 1). Hypoxaemia was identified in 102 of these patients (14%). Following final eligibility checks, 40 of these patients with hypoxaemia were excluded. Of 62 eligible patients approached to participate, 50 (81%) consented and were enrolled into the trial. Enrolment occurred on the first postoperative day for 30 participants (60%), second day for 14 (28%) and third day for 6 (12%). Enrolment ceased at the pre-determined sample size of 50 participants. Consent prior to enrolment was obtained directly from 41 patients and from the patient’s substitute decision maker in 5 cases. Consent following enrolment was required for four patients. One intervention group participant withdrew from the trial prior to receiving treatment. Primary outcome data were available for 100% of participants. Four participants were lost to the 90-day follow up.

#### Participant characteristics

Mean age was 62 (sd, 10) years. Full baseline characteristics are described in Table 1. No patient had hypoxaemia on hospital admission (SpO_2_ on room air; range, 93%–99%). On screening for entry into trial and detection of hypoxaemia, median respiratory rate was 17 (IQR, 16–18). During the first seven postoperative days, 40% of participants in the control group received HFOT and 33% in the intervention group (odds ratio [OR], 0.75; 95% confidence interval [CI], 0.20–2.80). The control group received 44 (sd, 26) h of HFOT and the intervention group received 54 (sd, 38) h (mean difference, 9; 95% CI, –41 to 22). Protocolised physiotherapy treatments were provided by 12 physiotherapists with expertise levels and number of protocol providers similar between treatment groups (Supplementary Table 4).

**Table 1:** Baseline characteristics. Values are mean (sd), median (IQR) or *n* (%) unless otherwise specified. ARISCAT, Assess Respiratory Risk in Surgical Patients in Catalonia; COPD, chronic obstructive pulmonary disease; DASI, Duke Activity Status Index; EQ5D, EuroQuol- 5D; FiO2, fraction of inspired oxygen; HFOT, high flow oxygen therapy; HPB, hepatopancrobiliary; IQR, interquartile range; OSA, obstructive sleep apnoea; ROX, respiratory rate – oxygenation; sd, standard deviation; UGI, upper gastrointestinal. ∗Updated.

	Control *n*=25	Intervention *n*=24
*Participant demographics*		
Age, yr (range)	44–81	38–78
BMI, kg/m^2^	31 (27–36)	30 (27–34)
Sex, male	10 (40)	15 (63)
Smoker	18 (72)	14 (58)
COPD	4 (16)	6 (25)
OSA	2 (8)	3 (13)
Cancer	8 (32)	13 (54)
Metastatic tumour	0 (0)	6 (25)
Anaemia	3 (12)	1 (4)
ROX on hospital admission	27 (3)	28 (4)
*Risk assessment*		
ASA>2	22 (88)	21 (88)
Charlson comorbidity index∗	1 (0–2)	2 (1–6)
ARISCAT medium-high risk	22 (88)	22 (92)
*Function and quality of life*		
Clinical frailty score	3 (1)	3 (1)
DASI	31 (16)	30 (15)
EQ5D		
Total score	8 (6–11)	7 (5–9)
VAS	60 (21)	71 (18)
*Surgical factors*		
Upper abdominal incision	16 (64)	17 (71)
Open incision	17 (68)	16 (67)
Elective surgery	19 (76)	18 (75)
Surgery duration, mins	282 (223–385)	303 (229–378)
Surgical specialty		
HPB/UGI	7 (28)	11 (46)
Colorectal	6 (24)	8 (33)
Urology/renal	5 (20)	3 (13)
Transplant	5 (20)	1 (4)
Vascular	2 (8)	1 (4)
Intraoperative tidal volumes, mls	490 (56)	526 (72)
Intraoperative FiO_2_	0.46 (0.08)	0.47 (0.08)
Intraoperative fluid, ml	2980 (1223–4100)	1570 (1350–5500)
*Postoperative*		
Nasogastric tube	9 (36)	11 (46)
Epidural	1 (4)	3 (13)
Location, ICU		
Immediately postoperative	5 (20)	7 (29)
At time of recruitment	1 (4)	5 (21)
HFOT within 3 h of extubation	5 (20)	5 (21)
HFOT at the time of screening	7 (28)	6 (25)
ROX at recruitment	20 (5)	20 (5)

### Primary outcome (feasibility)

#### Recruitment rate

Over 30 consecutive weeks, 50 participants were enrolled at a rate of 1.7 per week (95% CI, 0.7–3.4 participants per week).

#### Adherence to NIV protocol in the intervention group

In the 24 intervention group participants, 22 (92%) received physiotherapy-led NIV with a mean total duration delivered of 78 min (95% CI, 55–100) from a goal of 120 min. One participant refused all NIV sessions and did not complete any ‘Breathe’ or ‘Walk’ sessions to the protocol dose. The other participant had an undetected pneumothorax on trial enrolment. Prior to initiating NIV, a trial physiotherapist reviewed this participant’s chest imaging, detected the pneumothorax, and confirmed this with medical staff, thus contraindicating NIV. A per-protocol analysis removing these two participants observes a total mean NIV duration of 85 min (95% CI, 62–108).

Of 104 planned NIV sessions, it was unable to be delivered on 30 occasions (29%; 95% CI, 21%–38%). Reasons NIV was unable to be delivered or ceased prior to the 30-min goal dosage are described in Table 2. Of 74 NIV sessions initiated, the median NIV session length was 30 (IQR, 20–30) min and 69% (51/74; 95% CI, 58%–78%) were provided for the complete 30 min. On average, three NIV sessions (95% CI, 2–4) were delivered per participant. Eight additional sessions were delivered to four participants, most often for persistent hypoxaemia.

**Table 2:** Reasons for treatment non-adherence; sessions not delivered or not delivered to the goal dosage. Values are *n* (%). Proportions may not add up to 100% due to rounding. Between-group statistical analysis for non-adherence is displayed in Table 3. NIV, noninvasive ventilation.

Reasons	NIV		Breathe		Walk	
	Control No sessions	Intervention *n*=53 sessions	Control *n*=12 sessions	Intervention *n*=32 sessions	Control *n*=23 sessions	Intervention *n*=29 sessions
Pragmatic protocol non-adherence						
Declined – reason not given	-	16 (30)	6 (50)	12 (38)	3 (13)	10 (34)
Discomfort	-	8 (15)	0 (0)	3 (9)	2 (9)	0 (0)
Fatigue	-	7 (13)	0 (0)	2 (6)	5 (22)	2 (7)
Other priority	-	5 (9)	0 (0)	2 (6)	1 (4)	2 (7)
Pneumothorax prior to NIV	-	4 (8)	-	-	-	-
Pain	-	3 (6)	0 (0)	7 (22)	1 (4)	2 (7)
Participant unavailable	-	3 (6)	1 (8)	0 (0)	1 (4)	0 (0)
Nausea	-	2 (4)	3 (25)	1 (3)	1 (4)	3 (10)
Hypotension	-	2 (4)	0 (0)	0 (0)	2 (9)	0 (0)
Claustrophobia	-	2 (4)	-	-	-	-
Await test result	-	1 (2)	-	-	-	-
Drowsy	-	0 (0)	1 (8)	4 (13)	1 (4)	3 (10)
Participant unable to perform	-	0 (0)	1 (8)	0 (0)	0 (0)	0 (0)
Weekend – physio unavailable	-	-	-	-	4 (17)	6 (21)
Short of breath	-	-	-	-	1 (4)	0 (0)
Declined; independently walking	-	-	-	-	1 (4)	0 (0)
Dizzy	-	-	-	-	0 (0)	1 (3)
Low haemoglobin	-	-	-	-	0 (0)	0 (0)
Unintended protocol deviation		0 (0)	0 (0)	1 (3)	0 (0)	0 (0)

#### Acceptability of treatments

Detailed participant and clinician acceptability is reported in the supplement (Supplementary Tables 5a and 5b). For NIV, 20 of 22 patients who received NIV rated it as acceptable (91%; 95% CI, 72%–97%), although 27% (95% CI, 13%–48%) reported that NIV interfered with other priorities (Supplementary Fig. 1).

#### Safety

There were no serious adverse events in this trial. Overall adverse or transient physiological event rates during ‘Walk’ were 5% (8/164; 95% CI, 2%–9%) and NIV 4% (3/74; 95% CI, 1%–11%). No safety events were reported in relation to ‘Talk’ or ‘Breathe’ sessions.

Across all treatment modalities, transient physiological events occurred in 4/91 physiotherapy treatment sessions in control group participants (4%; 95% CI, 2%–11%) and 6/174 physiotherapy sessions in the intervention group participants (3%; 95% CI, 2%–7%); odds ratio [OR], 0.78 (95% CI, 0.21–2.83, *P*=0.70).

In the intervention group, one adverse event occurred during a ‘Walk’ session (1/83; 1%; 95% CI, 0.2%–7%), with symptomatic hypotension requiring medical review and fluid bolus. Six transient physiological events occurred: two incidences of hypotension during NIV, one episode of claustrophobia during NIV, two hypotensive episodes during mobilisation, and one episode of emesis during mobilisation.

In the control group, there were no adverse events and four transient physiological events: three episodes of hypotension and one of emesis during a ‘Walk’ session.

### Secondary outcomes

#### Trial enrolment and treatment milestones

Median time from diagnosis of hypoxaemia to group allocation was 75 min (IQR, 42–98) in the control group and 71 min (IQR, 56–106) in the intervention group. Time to first ambulation following surgery was 20 h (IQR, 17–33) in the control group and 21 h (IQR, 16–26) in the intervention group.

#### ‘Walk’ and ‘Breathe’ treatment adherence

Adherence to ‘Walk’ and ‘Breathe’ treatments is reported in Table 3. Pragmatic reasons ‘Walk’ and ‘Breathe’ treatments were not delivered are reported in Table 2. Additional ‘Breathe’ sessions were delivered in 14 of 20 planned sessions in the control group and 13 of 18 planned sessions in the intervention group, most often for persistent hypoxaemia.

**Table 3:** Walk and Breathe adherence. Values are median (interquartile range), *n* (%). ∗Odds ratio (95% confidence interval) or mean difference (95% confidence interval). ^†^Total number of sessions differed between treatment types and groups due to differences between patients in the number of days on protocol (Walk) and the number of additional sessions delivered (Breathe).

Treatments	Control	Intervention	Difference between groups*∗*
*Walk*			
Average number of sessions delivered per participant	3 (2–4)	3 (2–5)	0.22 (–0.50 to 0.94)
Total number of planned ambulation sessions^†^	92	94	
Session delivered full protocol dose	69 (75)	65 (69)	0.74 (0.38–1.42)
Session dose less than the protocol dose	12 (13)	18 (19)	1.58 (0.71–3.50)
Session not started	11 (12)	11 (12)	0.98 (0.40–2.38)
*Breathe*			
Average number of sessions delivered per participant	4 (4–5)	4 (2–5)	–0.52 (–1.40 to 0.36)
Total number of planned respiratory sessions^†^	120	114	
Session delivered full protocol dose	108 (90)	82 (72)	0.28 (0.14–0.59)
Session dose less than the protocol dose	6 (5)	15 (13)	2.88 (1.08–7.70)
Session not started	6 (5)	17 (15)	3.33 (1.26–8.78)

#### Unintended protocol deviations

Unintended protocol deviations occurred in four control and two intervention group participants. In the control group, two ‘Walk’ sessions exceeded 15 min, and two additional ‘Breathe’ sessions were unintentionally provided. In the intervention group, one ‘Breathe’ session was missed, and one extra ‘Breathe’ session was delivered unintentionally.

#### Exploratory clinical outcomes within groups

**Fig 2:**
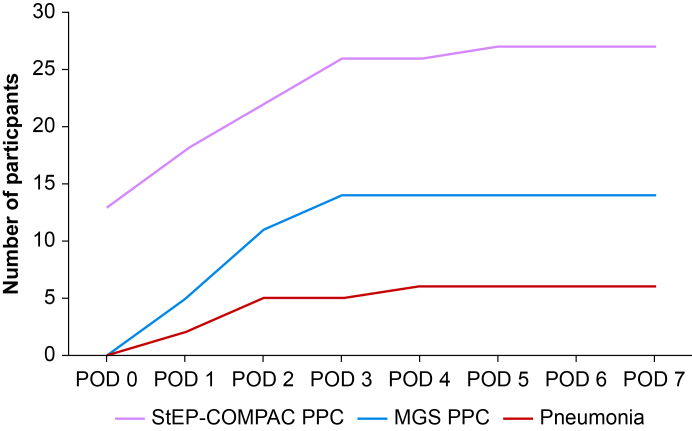
The cumulative number of participants with first day of diagnosis for each of three different PPC measurement tools. MGS, Melbourne Group Score; PPC, postoperative pulmonary complication; POD, postoperative day; StEP-COMPAC, Standardised End-Points in Perioperative medicine Core Outcome Measures for Perioperative and Anaesthetic Care.

The cumulative number of participants diagnosed with a PPC using three different PPC measurement tools across the first seven postoperative days is shown in Fig. 2. PPC incidence on trial enrolment ranged from 6% (3/49; 95% CI, 2%–17%) to 47% (23/49; 95% CI, 34%–61%). Chest imaging within the first seven postoperative days was ordered by the treating medical team in 82% (40/49) of participants. Radiologists reported atelectasis or collapse in 85% (34/40) of these participants. No participant required escalation to continuous NIV. Participants who required reintubation (6%; 3/49) or were readmitted to ICU (4%; 2/49) were for non-respiratory reasons.

#### Exploratory clinical outcomes between groups

**Fig 3:**
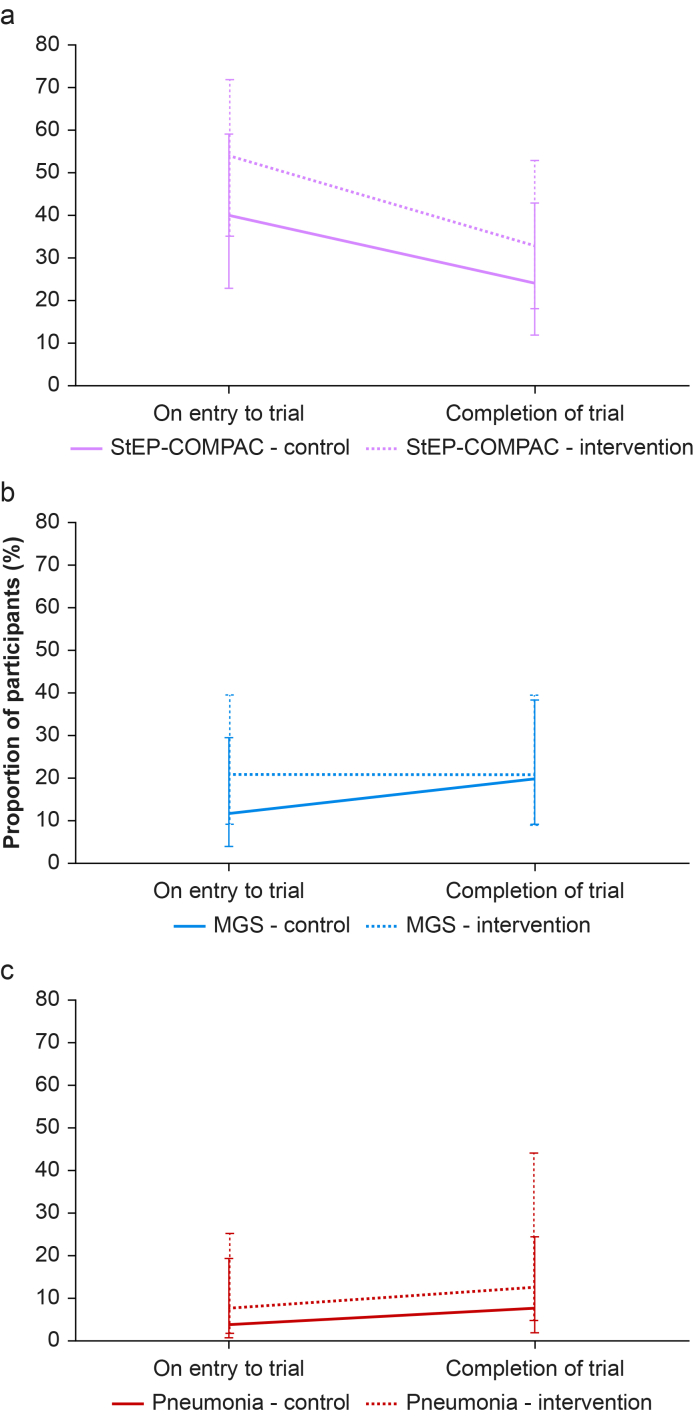
The incidence of three PPC measurement tools diagnosed on entry and at completion of the trial. a. StEP-COMPAC b. MGS c. Pneumonia. MGS, Melbourne Group Score PPC; Postoperative Pulmonary Complication; StEP-COMPAC, Standardised End-Points in Perioperative medicine Core Outcome Measures for Perioperative and Anaesthetic Care Error bars are 95% confidence intervals.

The between-group relative change in PPC incidence from the day of trial enrolment to trial completion for the three PPC diagnostic tools is displayed in Fig. 3. Change in peak cough flow and respiratory rate-oxygenation ratio immediately prior to and within 15 min of the first treatment session were similar between groups (Supplementary Table 6), as was time to resolution of hypoxaemia within the first seven postoperative days (Supplementary Fig. 2).

Length of stay, hospital re/admission, days alive and out of hospital and EQ-5D (Supplementary Tables 7 and 8) were similar between groups. In-hospital mortality occurred in one control group participant due to wound dehiscence and respiratory failure.

## Discussion

This single Center randomized pilot trial, a physiotherapist-led NIV using Air-Test-90 in patients with postoperative hypoxaemia, with an encouraging recruitment rate of approximately two participants per week. Physiotherapy and NIV treatments were well tolerated, acceptable, and successfully delivered to the majority of participants. The adverse event rate to physiotherapy-led NIV application was no higher than previously reported in similar cohorts.^16^^,^^24^

### Trial processes and recruitment

A similar recruitment rate to our study was observed in a single-centre physiotherapy trial of postoperative physiotherapy-led NIV,^16^ though the non-hypoxaemic cohort reported even higher consent rates (96%). Multicentre postoperative NIV studies^24^, ^25^, ^26^, ^27^, ^28^ have recruited large numbers of patients; however, when adjusted for the number of sites, recruitment rates were actually lower than our study. The PHYSIO+++ recruitment rate may reflect the study setting in a major quaternary centre with clinician investigators well established within the postoperative surgical environment, and possibly the consent model used. A hierarchical consent model^21^ permits enrolment of postoperative patients who otherwise may not be approached due to distress, cognition, or critical illness following surgery. A previous study enrolling patients following emergency laparotomy reported patient preference for consent following enrolment.^10^ Consent from a substitute decision maker or consent following enrolment was used for 20% of PHYSIO+++ participants, which likely enhanced both consent and recruitment rates, as well as reducing time from screening to recruitment (80 min) compared to a similar trial (3 h).^25^ Consent and recruitment processes within this single-centre pilot, therefore, appear feasible, and as an example, if a similar consenting model is followed and our hospital’s surgical volume and postoperative hypoxaemia incidence rate are generalisable to other centres, then a future trial run concurrently across 10 centres could recruit 10–30 eligible patients per week.

### Intervention delivery

In this study, we observed that once initiated, NIV sessions frequently achieved the planned dose, and in line with previous reports, missed NIV sessions accounted for the majority of NIV time lost.^16^ Thus, NIV session initiation appears to be key to adherence, however, it may be limited without access to suitably trained staff. Physiotherapy staff availability limited NIV adherence in a previous study,^16^ however this was infrequently a barrier in our large quaternary hospital setting with a 7-day physiotherapy service. The acceptability of treatments to participants is also known to limit adherence.^16^^,^^25^ In our study, despite high general acceptability (91%), 27% of participants reported that the NIV intervention interfered with other priorities of their postoperative recovery, and 20% found it uncomfortable. However, notably, the effort of NIV was rated lower than the effort of walking, which may explain reported low adherence to early mobility in surgical studies, despite recommendations in Early Recovery After Surgery protocols.^29^ For NIV adherence, incomplete dose delivery may not infer a lack of therapeutic value. A medical-led trial of adults in ICU with postoperative hypoxaemic respiratory failure reported a reduction in reintubation rates in the NIV group despite 31% of patients not receiving the goal NIV dose.^25^ Adherence to NIV protocols is a recognised limitation, irrespective of profession delivering the therapy.^16^^,^^24^, ^25^, ^26^, ^27^, ^28^ Strategies to improve NIV session initiation and identification of an optimal NIV dose should be a focus in future trials.

### Safety of intervention delivery

Postoperative nausea and hypotension are common side effects following major surgery.^30^ Mild adverse events, including hypotension and nausea, were almost always ameliorated with cessation of therapy, which suggests that our trial assessment and monitoring processes were timely and appropriate. We report similar rates of adverse events to previous studies of postoperative physiotherapist-led NIV.^16^^,^^28^ Routine early mobility and NIV in selected groups continue to be recommended,^12^^,^^13^^,^^29^ despite the risk of postoperative side effects, possibly due to reported infrequent adverse events and the potential benefits of treatments, including avoidance of more serious complications. Monitoring for serious adverse events, safety stopping rules and Data Safety Monitoring required for larger studies would provide additional safety assurance.

### Exploring outcome measure selection for future trials

Due to our novel inclusion criteria (hypoxaemia diagnosed with the Air-Test-90), we investigated three PPC tools as potential outcome measures for a definitive trial. Considering our observed PPC incidence (47%) prior to trial enrolment, recruitment rates in a future trial would be reduced considerably if patients with a PPC diagnosis were excluded. Alternately, a future effectiveness trial could use repeated PPC measures or severity scales to preserve recruitment rates and add to the limited available evidence for physiotherapy treatment of patients diagnosed with a PPC. Studies testing NIV to treat postoperative hypoxaemia often target recruitment of patients with concurrent acute respiratory failure^31^ in higher-acuity locations.^25^, ^26^, ^27^, ^28^ In our study, the Air-Test-90 identified patients without concurrent respiratory failure (respiratory rate 17 breaths min^−1^). Therefore, if uncommon at the time of enrolment, respiratory failure may be an ideal primary outcome measure for a future effectiveness study. Alternatively, a composite respiratory failure measure, similar to previous studies,^24^ could be chosen to leverage the combined higher outcome incidence and reduce sample size requirements.

### Strengths and limitations

Strengths of the PHYSIO+++ trial include the accessible approach to hypoxaemia diagnosis (Air-Test-90) and care location (including surgical wards), and permitting inclusion of patients with an existing PPC diagnosis. Limitations include that hypoxaemia may have been missed if it occurred outside the Air-Test-90 assessment time. Also, we did not specify all patients were to be awake during the hypoxaemia assessment, which may have increased heterogeneity. As a pragmatic trial, we did not protocolise HFOT, however, this allowed us to describe current HFOT usage for this population at our hospital. Participants and some clinicians were not blinded, and the success of assessor blinding was not evaluated, which may be a source of bias. Generalisability of our trial is also limited to sites where NIV can be provided by physiotherapists, including on weekends. Lastly, we did not predefine feasibility thresholds or progression criteria due to the external nature of our pilot study, and our small sample size limited the certainty of feasibility estimates.

### Implications for future study trial design and progression to a future trial

Elements of the current protocol that should remain unchanged in a future study include the recruitment and consent process and NIV session duration (30 min). However, while recruitment rates observed within a tertiary hospital were encouraging, these rates may not be generalisable within a multicentre study. Elements that require modification include the selection of outcome measures if a future definitive trial aims to test NIV effectiveness, which may require a composite of respiratory failure measures. In addition, the logistics of NIV delivery may require modification to improve rates of session initiation, as it is uncertain whether the observed NIV adherence levels are sufficient for scaling to a definitive trial. Therefore, further study is required to identify an optimal therapeutic NIV dose given participant acceptability, suggesting preference for fewer sessions to avoid interference with other priorities. Feasibility challenges included a high screening burden to confirm surgical eligibility, however electronic medical records facilitated this process.

## Conclusions

Recruitment to a trial of physiotherapist-led NIV in addition to usual care physiotherapy was feasible, observing high participant NIV acceptability and similar adherence parameters as previous trials for adults with hypoxaemia following abdominal surgery. PHYSIO+++ is the first physiotherapy study to enrich recruitment using the Air-Test-90 and provides valuable insights for the design and conduct of future postoperative physiotherapy treatment studies.

## Authors’ contributions

Funding acquisition, project administration, writing- original draft preparation: CH

Conceptualisation: CH, IB

Writing – review and editing: IB, LD, PK, NRe, NRi, SF, BMS

Methodology: CH, IB, LD, PK

Formal analysis: NRe

Data curation: NRi, SF

Investigation: CH, NRi, SF

Software: SF

## Data availability

The datasets generated and/or analysed during the current trial are not publicly available, however are available on reasonable request. Requests for data may be sent to the corresponding author at claire.hackett@health.qld.gov.au.

## Funding

This work was supported by Metro South Research Support Scheme 2022 (grant number RSS2022_034). The funding organisation had no role in trial design, collection, management, analysis, or interpretation of the data or written reports. This research was supported by the Commonwealth through an Australian Government Research Training Program Scholarship (DOI: https://doi.org/10.82133/C42F-K220).

## Declaration of interest

The authors declare that they have no conflicts of interest.
