# Supplementary material for: Physiotherapist-led intermittent noninvasive ventilation for hypoxaemia following abdominal surgery in a quaternary Australian hospital: a randomised pilot feasibility trial
Source: BJA Open. 2026 Jul 13;19:100571. doi: 10.1016/j.bjao.2026.100571 (PMC13382445; doi:10.1016/j.bjao.2026.100571)
Supplement: multimedia component 1 [file mmc1.docx]

Online Supplementary File S1: PHYSIO+++ Supplementary materials

**Table S1: CONSORT checklist for PHYSIO+++**

| **Section/Topic** | **No** | **Original CONSORT 2010^1^ + Pilot/Feasibility^2^ checklist item** | **Non-pharmacologic Trials^3^** | **Pragmatic trials^4^** |
| --- | --- | --- | --- | --- |
| Title and abstract | 1a | Identification as a pilot or feasibility randomised trial in the title  Title - Page 1-2 | In the abstract, description of the experimental treatment, comparator, care providers, centres, and blinding status.  Page 2 |  |
|  | 1b | Structured summary of pilot trial design, methods, results, and conclusions  Page 2 |  |  |
| Introduction  Background and objectives | 2a | Scientific background and explanation of rationale for future definitive trial, and reasons for randomised pilot trial  Page 3-4 |  | Describe the health or health service problem that  the intervention is intended to address and other  interventions that may commonly be aimed at this problem  Page 3-4 |
|  | 2b | Specific objectives or research questions for pilot trial  Page 4 |  |  |
| Methods  Trial design | 3a | Description of pilot trial design (such as parallel, factorial) including allocation ratio  Page 5 and Protocol paper |  |  |
|  | 3b | Important changes to methods after pilot trial commencement (such as eligibility criteria), with reasons  Supplementary Table S3 |  |  |
| Participants | 4a | Eligibility criteria for participants  Page 5 and Supplementary Table S2 | When applicable, eligibility criteria for centres and those performing the interventions  Page 5-6 | Eligibility criteria should be explicitly framed to show  the degree to which they include typical participants  and/or, where applicable, typical providers (eg,  nurses), institutions (eg, hospitals), communities (or  localities eg, towns) and settings of care (eg, different healthcare financing systems)  Page 5-6. Supplementary Table S2 |
|  | 4b | Settings and locations where the data were collected  Page 4-5 and Protocol paper |  |  |
|  | 4c |  | How participants were identified and consented  Page 5 and Protocol paper |  |
| Interventions | 5 | The interventions for each group with sufficient details to allow replication, including how and when they were actually administered  Page 6-7 and Protocol paper | A: Precise details of both the experimental treatment and comparator  B: Description of the different components of the interventions and, when applicable, descriptions of the procedure for tailoring the interventions to individual participants  C: Details of how the interventions were standardized  D: Details of how adherence of care providers with the protocol was assessed or enhanced  Page 6-7 and Protocol paper | Describe extra resources added to (or resources removed from) usual settings in order to implement intervention. Indicate if efforts were made to standardise the intervention or if the intervention and its delivery were allowed to vary between participants, practitioners, or study sites  Describe the comparator in similar detail to the intervention  Page 6-7 and Protocol paper |
| Outcomes | 6a | Completely defined pre-specified assessments or measurements to address each pilot trial objective specified in 2b, including how and when they were assessed.  Pages 7-8 and Protocol paper |  | Explain why the chosen outcomes and, when relevant, the length of follow-up are considered important to those who will use the results of the trial  Protocol paper |
|  | 6b | Any changes to pilot trial assessments after the pilot trial commenced, with reasons  Supplementary Table S3 |  |  |
|  | 6c | If applicable, prespecified criteria used to judge whether or how to proceed with future definitive trial.  Page 18 |  |  |
| Sample size | 7a | Rationale for numbers in the pilot trial  Page 9 and Protocol paper | When applicable, details of whether and how the clustering by care providers or centres was addressed  Page 6 and Supplementary Table 3 | If calculated using the smallest difference considered important by the target decision maker audience (the minimally important difference) then report where this difference was obtained  N/A |
|  | 7b | When applicable, explanation of any interim analyses and stopping guidelines  N/A |  |  |
| Randomisation  Sequence  generation | 8a | Method used to generate the random allocation sequence  Page 5-6 and Protocol paper | When applicable, how care providers were allocated to each trial group  Page 6 |  |
|  | 8b | Type of randomisation; details of any restriction (such as blocking and block size)  Page 6. Protocol paper |  |  |
| Allocation concealment mechanism | 9 | Mechanism used to implement the random allocation sequence (such as sequentially numbered containers), describing any steps taken to conceal the sequence until interventions were assigned  Page 5-6. Protocol paper |  |  |
| Implementation | 10 | Who generated the random allocation sequence, who enrolled participants, and who assigned participants to interventions  Page 5-6. Protocol paper |  |  |
| Blinding | 11a | If done, who was blinded after assignment to interventions (for example, participants, care providers, those assessing outcomes) and how  Page 8 and Protocol paper | Whether or not those administering co-interventions were blinded to group assignment  Page 8 and Protocol paper | If blinding was not done, or was not possible, explain why  Page 8 and Protocol paper |
|  | 11b | If relevant, description of the similarity of interventions  Page 6-7 and protocol paper | If blinded, method of blinding and description of the similarity of interventions.  Page 7-8 and Protocol paper |  |
| Statistical methods | 12a | Statistical methods used to compare groups for primary and secondary outcomes  Methods used to address each pilot toral objective whether qualitative or quantitative  Page 9 | When applicable, details of whether and how the clustering by care providers or centres was addressed  N/A |  |
|  | 12b | Methods for additional analyses, such as subgroup analyses and adjusted analyses  Page 8 |  |  |
| Results  Participant flow (a diagram is strongly recommended) | 13a | For each group, the numbers of participants who were approached and/or assessed for eligibility, randomly assigned, received intended treatment, and were assessed for each objective  Page 10, Figure 1- Consort flow diagram | The number of care providers or centres performing the intervention in each group and the number of patients treated by each care provider or in each centre  Supplementary Table S4 | The number of participants or units approached to take part in the trial, the number which were eligible, and reasons for non-participation should be reported  Figure 1 - Consort Flow diagram |
|  | 13b | For each group, losses and exclusions after randomisation, together with reasons  Page 10, Figure 1 - Consort flow diagram | b. Details of the experimental treatment and comparator as they were implemented  c. For each group, the delay between randomisation and the initiation of the intervention.  Page 10-14. Protocol paper |  |
| Recruitment | 14a | Dates defining the periods of recruitment and follow-up  Page 5 |  |  |
|  | 14b | Why the pilot trial ended or was stopped  Page 10 |  |  |
| Baseline data | 15 | A table showing baseline demographic and clinical characteristics for each group  Table 1 | When applicable, a description of care providers (case volume, qualification, expertise, etc.) and centres (volume) in each group  Supplementary Table S4 |  |
| Numbers analysed | 16 | For each objective, number of participants (denominator) included in each analysis. If relevant, these numbers should be by randomised group  Page 10-14, Supplementary materials |  |  |
| Outcomes and estimation | 17a | For each objective, results including expressions of uncertainty (such as 95% confidence interval) for any  estimates. If relevant, these results should be by randomised group  Page 11-17 |  |  |
|  | 17b | For binary outcomes, presentation of both absolute and relative effect sizes is recommended  Page 11-14 |  |  |
| Ancillary analyses | 18 | Results of any other analyses performed, including subgroup analyses and adjusted analyses, distinguishing pre-specified from exploratory  N/A |  |  |
| Harms | 19 | All important harms or unintended effects in each group (for specific guidance see CONSORT for harms)  a. If relevant, other important unintended consequences  Page 12 |  |  |
| Discussion  Limitations | 20 | Pilot trial limitations, addressing sources of potential bias and remaining uncertainty about feasibility  Page 15-18 | In addition, take into account the choice of the comparator, lack of or partial blinding, and unequal expertise of care providers or centres in each group  Page 15-18 |  |
| Generalisability | 21 | Generalisability (applicability) of pilot trial methods and findings to future definitive trial and other studies  Page 17-18 | Generalizability (external validity) of the trial findings according to the intervention, comparators, patients, and care providers and centres involved in the trial  Page 18 | Describe key aspects of the setting which determined the trial results. Discuss possible differences in other settings where clinical traditions, health service organisation, staffing, or resources may vary from those of the trial  Page 15-18 |
| Interpretation | 22 | Interpretation consistent with pilot trial objectives and findings, balancing potential benefits and harms, and  considering other relevant evidence  a. Implications for progression from pilot to future definitive trial, including any proposed amendments.  Page 15-18 | In addition, take into account the choice of the comparator, lack of or partial blinding, and unequal expertise of care providers or centres in each group  Page 15- 18 |  |
| Other information Registration | 23 | Registration number for pilot trial and name of trial registry  Page 5 |  |  |
| Protocol | 24 | Where the pilot trial protocol can be accessed, if available  Reference list #17 in manuscript |  |  |
| Funding | 25 | Sources of funding and other support (such as supply of drugs), role of funders  Page 22 |  |  |
|  | 26 | Ethical approval or approval by research review committee, confirmed with reference number  Page 1,5 |  |  |

**References:**

1. Schulz KF, Altman DG, Moher D, Group C. CONSORT 2010 statement: updated guidelines for reporting parallel group randomised trials. BMJ. 2010;340:c332. Eldridge SM, Chan CL, Campbell MJ, Bond CM, Hopewell S, Thabane L, et al. CONSORT 2010 statement: extension to randomised pilot and feasibility trials. BMJ. 2016;355.
2. Boutron I, Altman DG, Moher D, Schulz KF, Ravaud P, Group CN. CONSORT Statement for Randomized Trials of Nonpharmacologic Treatments: A 2017 Update and a CONSORT Extension for Nonpharmacologic Trial Abstracts. Ann Intern Med. 2017;167(1):40-7.
3. Zwarenstein M, Treweek S, Gagnier JJ, Altman DG, Tunis S, Haynes B, et al. Improving the reporting of pragmatic trials: an extension of the CONSORT statement. BMJ. 2008;337:a2390.

**Table S2:** PHYSIO+++ eligibility and exclusion criteria

| Eligibility | - Major elective or emergency abdominal surgery via an open (≥5cm), laparoscopic or robotic abdominal incision with an anaesthetic time ≥ 3 hours - Extubated within 24 hours of surgery completion - Breathing without - Age ≥ 18 years at time of surgery - Hypoxaemia at least 3 hours after extubation and within 72 hours of surgery |
| --- | --- |
| Exclusions | - Non-consent to participate - Unable to understand English without an interpreter - Severe cognitive impairment - Pregnancy - Oesophagectomy - Presence of a tracheostomy or other artificial airway - Previously participated in PHYSIO+++ - Current enrolment in a trial with similar treatments or outcomes - Patients under airborne or droplet precautions - Premorbid neuromuscular condition with significant muscle weakness necessitating manual or mechanical assistance to cough - Not able to be recruited within 8 hours of being assessed as eligible to enter the trial - NIV or CPAP for premorbid sleep disordered breathing utilised during hospital admission - Does not receive medical clearance to participate in the trial due to:  1. Imminent (anticipated within 12 hours of study inclusion) surgery, palliation, reintubation or the need for continuous medically prescribed NIV/CPAP 2. Profound respiratory failure or cardiovascular instability 3. NIV contraindicated (Hackett 2023 Supplementary Table 1) |

CPAP, continuous positive airway pressure support; NIV, non-invasive ventilation

**Table S3:** Changes to methods and outcome measures following trial commencement

| Change | Reason |
| --- | --- |
| Methods | |
| Addition of oesophagectomy and home oxygen to exclusion criteria | While NIV has been utilised safely following oesophagectomy in ICU trials to treat hypoxaemia, we excluded oesophagectomy due to the reduced monitoring available on surgical wards |
| Participant acceptability questionnaire format change | To improve readability |
| Clinician acceptability questionnaire extended from only those providing therapies to trial participants to all physiotherapists at the trial site who provide care to patients undergoing abdominal surgery | Low numbers of physiotherapists providing therapy to trial participants may result in unblinding of clinician participants. |
| Ward physiotherapist case report form: Peak cough flow measure increase from one to three measures taken | To encourage recording of three peak cough flow measures at a time point and subsequent selection of the best of three in line with usual practise for this measure |
| Ward physiotherapist case report form: Post therapy data point added for immediately post treatment session completion (i.e. physiotherapist leaving the bedside) using the post therapy measure from the last provided treatment (i.e. Walk, Breathe or NIV) in addition to existing 15-minute post time point | Patients were often not available (off ward, in bathroom, with other staff) at the 15-minutes post time frame. Using the last measure before the physiotherapist left the bedspace created an alternate post therapy measure. |
| Outcome measures | |
| The primary measure of NIV adherence was altered during the trial to better describe NIV adherence to the planned 120-minutes minimum dose as the minimum clinically meaningful dose in this population is unknown. | This outcome measure will describe the adherence to the physiotherapist-led NIV set protocolised dosage of a minimum of 4 sessions (120-minutes) rather than the previously described outcome of minimum 30-minutes dose. |
| Clarification of length of stay and in-hospital mortality timeframe to 90 days at most | Clarification to the timeframe to align with the follow up period for this study. |
| Daily incidence of persistent hypoxaemia | This outcome will assess response to therapy. |
| Change to severity of MGS PPC and Independent Pneumonia outcome measures | These outcome measures will enable further exploration of PPC diagnosis and progress. |
| Incidence and reason for hospital readmission at 30 and 90 postop days | Had not been listed as a planned outcome though was included in data collection tools |
| Mortality at 30 postop days | Consistency of measures at follow up time frames 30 and 90 postop days |
| Removal of CFS as an outcome measure at 90 postoperative days | CFS will be reported at baseline, though trial therapies are not anticipated to impact on CFS at 90 postop days. |

CFS, Clinical Frailty Score, ICU, Intensive Care Unit; MGS, Melbourne Group Score; NIV, Non-invasive ventilation; PPC, Postoperative Pulmonary Complication,

**Table S4:** Description of care providers (case volume, qualification, expertise)

|  | Physiotherapist expertise | Active control | Intervention |
| --- | --- | --- | --- |
| Number of physiotherapists providing care (n) |  | 6 | 7 |
| Number of participants provided care by each physiotherapist (n)  Physiotherapist 1  Physiotherapist 2  Physiotherapist 3  Physiotherapist 4  Physiotherapist 5  Physiotherapist 6  Physiotherapist 7  Physiotherapist 8  Physiotherapist 9  Physiotherapist 10  Physiotherapist 11  Physiotherapist 12 | Expert  Intermediate  Senior  Novice  Novice  Expert  Expert  Senior  Novice  Novice  Novice  Intermediate | 17  15  3  1  1  0  1  0  0  0  1  0 | 19  15  0  1  0  1  0  1  1  1  0  1 |

Definition of expertise as Boden et al 2024

Novice: <2years experience

Intermediate: 2 to 5 years of experience

Senior: 5 to 10 years of experience

Expert: >10 years of experience

*Boden I, Reeve J, Jernas A, Denehy L, Fagevik Olsen M. Preoperative physiotherapy prevents postoperative pulmonary complications after major abdominal surgery: a meta-analysis of individual patient data. J Physiother. 2024;70(3):216-23.*


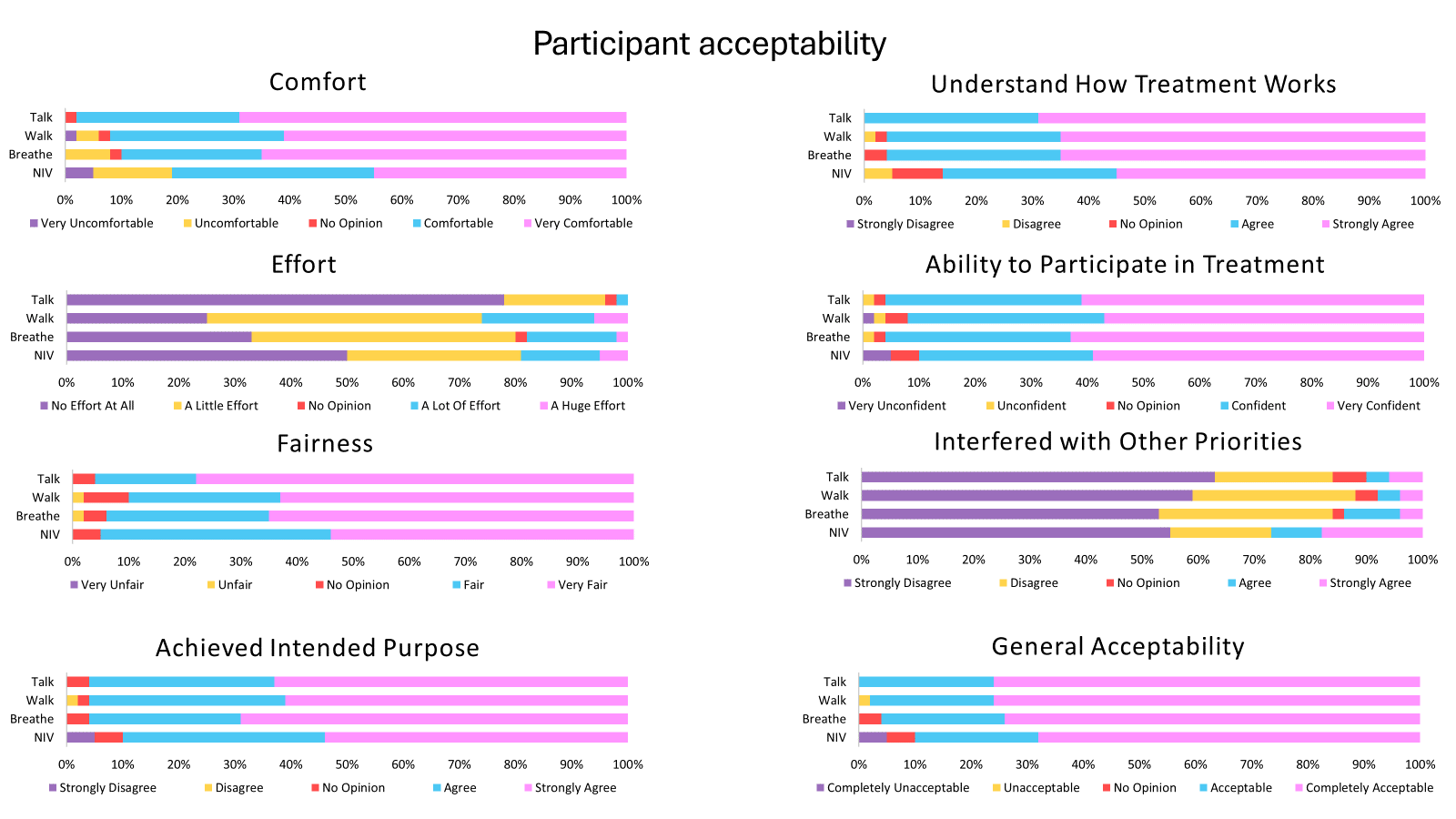


**Figure S1** – Participant acceptability questionnaire results

*NIV, Noninvasive ventilation. Participants in the intervention group who did not receive NIV (n=2) were included in the intention to treat analysis, however, did not provide responses to acceptability questions for NIV.*

**Clinician acceptability questionnaire**

Members of the research team were not eligible to complete the clinician acceptability questionnaire due to risk of bias. The number of clinicians who responded to the questionnaire after providing any trial therapy was small (n=7) which may have led to unblinding responses. As a result, the questionnaire was sent to all Physiotherapists who work on abdominal surgical wards at our site. For those clinicians who did not provide protocol therapy to trial participants, responses were requested to relate to their previous experience providing Talk, Walk, Breathe and NIV to patients following abdominal surgery. A total of 15 clinicians completed the questionnaire. A second response at the end of trial was provided by two clinicians. This data was not analysed due to the risk of unblinding these two clinicians and the low volume of second responses.

Median years of experience for clinicians was 8 (IQR 2-11.5) years. NIV had never been provided to any patient following abdominal surgery by six (40%) and walk not provided by one clinician/s (7%). One clinician responded Yes to having provided NIV in the pre-acceptability questions (Supplementary Table 4a) however selected “Never provided” in the acceptability questionnaire (Supplementary Table 4b) for each NIV question. Two clinicians selected “Never provided” NIV in the pre-acceptability questions however provided a response to the acceptability NIV questions. One clinician answered “Yes” to providing Walk in the pre-acceptability questions, however selected “Never provided” in the acceptability questionnaire for each of the Walk questions.

Clinicians reported conflicting responses in the pre-questionnaire and the acceptability questionnaire which may reflect the structure of the questionnaire tool or the unsupervised method of questionnaire distribution (via email with link to REDCap). Automated removal of questions based on the pre-questionnaire responses may avoid similar results in future.

Flipped responses, where the less positive response moves from the furthest right to the furthest left and double negative question/answers (for example the Opportunity costs domain) may result in unintended responses if the clinician/participant does not closely read the question and possible responses. For these same reasons, we found participants required supervision when undertaking the questionnaire to identify questions with flipped responses or double negatives.

The clinician questionnaire should not be replicated in its current form. Participants should be supervised during questionnaire completion.

**Table S5a** - Clinician pre-questionnaire responses

|  | Yes | No |
| --- | --- | --- |
| Have you provided the protocolised "Talk" therapy to a participant enrolled in the PHYSIO+++ trial? | 3 (20%) | 12 (80%) |
| Have you provided the protocolised "Walk" therapy to a participant enrolled in the PHYSIO+++ trial? | 5 (33%) | 10 (67%) |
| Have you provided the protocolised "Breathe" therapy to a participant enrolled in the PHYSIO+++ trial? | 7 (47%) | 8 (53%) |
| Have you provided the protocolised "NIV" therapy to a participant enrolled in the PHYSIO+++ trial? | 1 (7%) | 14 (93%) |
| Have you provided information to an adult following their abdominal surgery? | 15 (100%) | 0 (0%) |
| Have you facilitated walking or other physical exercises to an adult following their abdominal surgery? | 15 (100%) | 0 (0%) |
| Have you coached breathing exercises to an adult following their abdominal surgery? | 15 (100%) | 0 (0%) |
| Have you provided NIV to an adult following their abdominal surgery? | 10 (67%) | 5 (33%) |
| For all of the following questions please respond in relation to the protocolised therapy that you have delivered to participants enrolled in the PHYSIO+++ trial. If you have not delivered any one of the therapies (Talk, Walk, Breathe, NIV) to an enrolled PHYSIO+++ participant, please instead respond based on your previous experience of delivering the therapy to adults following their abdominal surgery. I understand and will follow this instruction. | 15 (100%) | 0 (0%) |

**Table S5b** – Clinician acceptability raw questionnaire responses

| Acceptability Domains | Clinician Responses n (%) | | | | | |
| --- | --- | --- | --- | --- | --- | --- |
| *Affective attitude. How an individual feels about the intervention.* | Very Uncomfortable | Uncomfortable | No Opinion | Comfortable | Very Comfortable | Never  Provided |
| How comfortable did you feel providing information? | 1 (7%) | 0 (0%) | 0 (0%) | 7 (47%) | 7 (47%) | 0 (0%) |
| How comfortable did you feel facilitating walking or other physical exercises? | 1 (7%) | 0 (0%) | 0 (0%) | 4 (27%) | 9 (60%) | 1 (7%) |
| How comfortable did you feel coaching breathing exercises? | 1 (7%) | 0 (0%) | 0 (0%) | 4 (27%) | 10 (67%) | 0 (0%) |
| How comfortable did you feel providing NIV? | 1 (7%) | 1 (7%) | 1 (7%) | 5 (33%) | 4 (27%) | 3 (20%) |
| *Burden. The amount of effort required to participate in the intervention.* | No Effort  At All | A Little  Effort | No Opinion | A Lot of  Effort | A Huge  Effort | Never  Provided |
| How much effort did it take to provide information? | 7 (47%) | 7 (47%) | 0 (0%) | 1 (7%) | 0 (0%) | 0 (0%) |
| How much effort did it take to facilitate walking or other physical exercise? | 1 (7%) | 11 (73%) | 0 (0%) | 2 (13%) | 0 (0%) | 1 (7%) |
| How much effort did it take to coach breathing exercises? | 3 (20%) | 12 (80%) | 0 (0%) | 0 (0%) | 0 (0%) | 0 (0%) |
| How much effort did it take to provide NIV? | 0 (0%) | 6 (40%) | 3 (20%) | 3 (20%) | 0 (0%) | 3 (20%) |
| *Ethicality. The extent to which the intervention has good fit with an individual's value system* | Very  Unfair | Unfair | No Opinion | Fair | Very  Fair | Never  Provided |
| How fair was it to provide information? | 0 (0%) | 0 (0%) | 0 (0%) | 5 (33%) | 10 (67%) | 0 (0%) |
| How fair was it to facilitate walking or other physical exercise? | 0 (0%) | 0 (0%) | 0 (0%) | 3 (20%) | 11 (73%) | 1 (7%) |
| How fair was it to coach breathing exercises? | 0 (0%) | 0 (0%) | 0 (0%) | 6 (40%) | 9 (60%) | 0 (0%) |
| How fair was it to provide NIV? | 0 (0%) | 0 (0%) | 1 (7%) | 6 (40%) | 5 (33%) | 3 (20%) |
| *Perceived effectiveness. The extent to which the intervention is perceived to have achieved its intended purpose.* | Strongly  Disagree | Disagree | No Opinion | Agree | Strongly  Agree | Never  Provided |
| Providing information achieved its purpose. | 0 (0%) | 1 (7%) | 1 (7%) | 7 (47%) | 6 (40%) | 0 (0%) |
| Facilitating walking or other physical exercises achieved its purpose. | 0 (0%) | 0 (0%) | 0 (0%) | 4 (27%) | 10 (67%) | 1 (7%) |
| Coaching breathing exercises achieved its purpose. | 0 (0%) | 1 (7%) | 1 (7%) | 9 (60%) | 4 (27%) | 0 (0%) |
| Providing NIV achieved its purpose. | 0 (0%) | 0 (0%) | 2 (13%) | 7 (47%) | 3 (20%) | 3 (20%) |

| Acceptability Domains | Clinician Responses n (%) | | | | | |
| --- | --- | --- | --- | --- | --- | --- |
| *Intervention coherence. The extent to which the clinician understands how the intervention works* | Strongly  Disagree | Disagree | No Opinion | Agree | Strongly  Agree | Never  Provided |
| It is clear how providing information will or has helped improve their recovery. | 0 (0%) | 1 (7%) | 0 (0%) | 9 (60%) | 5 (33%) | 0 (0%) |
| It is clear how facilitating walking or other physical exercise will or has helped to improve their recovery. | 0 (0%) | 0 (0%) | 0 (0%) | 5 (33%) | 9 (60%) | 1 (7%) |
| It is clear how coaching breathing exercises will or has helped to improve their recovery. | 0 (0%) | 1 (7%) | 1 (7%) | 6 (40%) | 7 (47%) | 0 (0%) |
| It is clear how providing NIV will or has helped to improve their recovery. | 0 (0%) | 0 (0%) | 3 (20%) | 5 (33%) | 4 (27%) | 3 (20%) |
| *Self-efficacy. The clinician’s confidence that they can perform behaviour(s) required to participate in the intervention.* | Very  Unconfident | Unconfident | No Opinion | Confident | Very  Confident | Never  Provided |
| How confident did you feel about being able to provide information? | 0 (0%) | 0 (0%) | 0 (0%) | 8 (53%) | 7 (47%) | 0 (0%) |
| How confident did you feel about facilitating walking or other physical exercises? | 0 (0%) | 0 (0%) | 0 (0%) | 5 (33%) | 9 (60%) | 1 (7%) |
| How confident did you feel about coaching breathing exercises? | 0 (0%) | 0 (0%) | 0 (0%) | 8 (53%) | 7 (47%) | 0 (0%) |
| How confident did you feel about providing NIV? | 0 (0%) | 2 (13%) | 2 (13%) | 3 (20%) | 5 (33%) | 3 (20%) |
| *Opportunity costs. The benefits, profits or values that were given up to engage in the intervention.* | Strongly  Disagree | Disagree | No Opinion | Agree | Strongly  Agree | Never  Provided |
| Providing information interfered with my other priorities. | 2 (13%) | 8 (53%) | 1 (7%) | 3 (20%) | 1 (7%) | 0 (0%) |
| Facilitating walking or other physical exercise interfered with my other priorities. | 3 (20%) | 7 (47%) | 0 (0%) | 2 (13%) | 2 (13%) | 1 (7%) |
| Coaching breathing exercises interfered with my other priorities. | 2 (13%) | 9 (60%) | 1 (7%) | 2 (13%) | 1 (7%) | 0 (0%) |
| Providing NIV interfered with my other priorities. | 1 (7%) | 3 (20%) | 5 (33%) | 2 (13%) | 1 (7%) | 3 (20%) |
| *General Acceptability* | Completely Unacceptable | Unacceptable | No Opinion | Acceptable | Completely Acceptable | Never  Provided |
| How acceptable was providing information? | 0 (0%) | 0 (0%) | 0 (0%) | 5 (33%) | 10 (67%) | 0 (0%) |
| How acceptable was facilitating walking or other physical exercise? | 0 (0%) | 0 (0%) | 0 (0%) | 4 (27%) | 10 (67%) | 1 (7%) |
| How acceptable was coaching breathing exercises? | 0 (0%) | 0 (0%) | 0 (0%) | 7 (47%) | 8 (53%) | 0 (0%) |
| How acceptable was providing NIV? | 0 (0%) | 0 (0%) | 3 (20%) | 5 (33%) | 4 (27%) | 3 (20%) |
| *Note: Questionnaire questions have been abbreviated in this table. Please refer to the protocol^19^ for full question format. Data may not add up to 100% due to rounding.* | | | | | | |

*NIV, Non-invasive ventilation*

**Table S6:** Between group comparison of Peak cough flow and ROX index pre and post treatment on the day of enrolment

|  | Coefficient change | 95% CI | p |
| --- | --- | --- | --- |
| ROX | 1.42 | -1.69 to 4.51 | 0.36 |
| Peak cough flow | -1.59 | -46.38 to 43.19 | 0.94 |

*ROX, Respiratory rate- oxygenation*


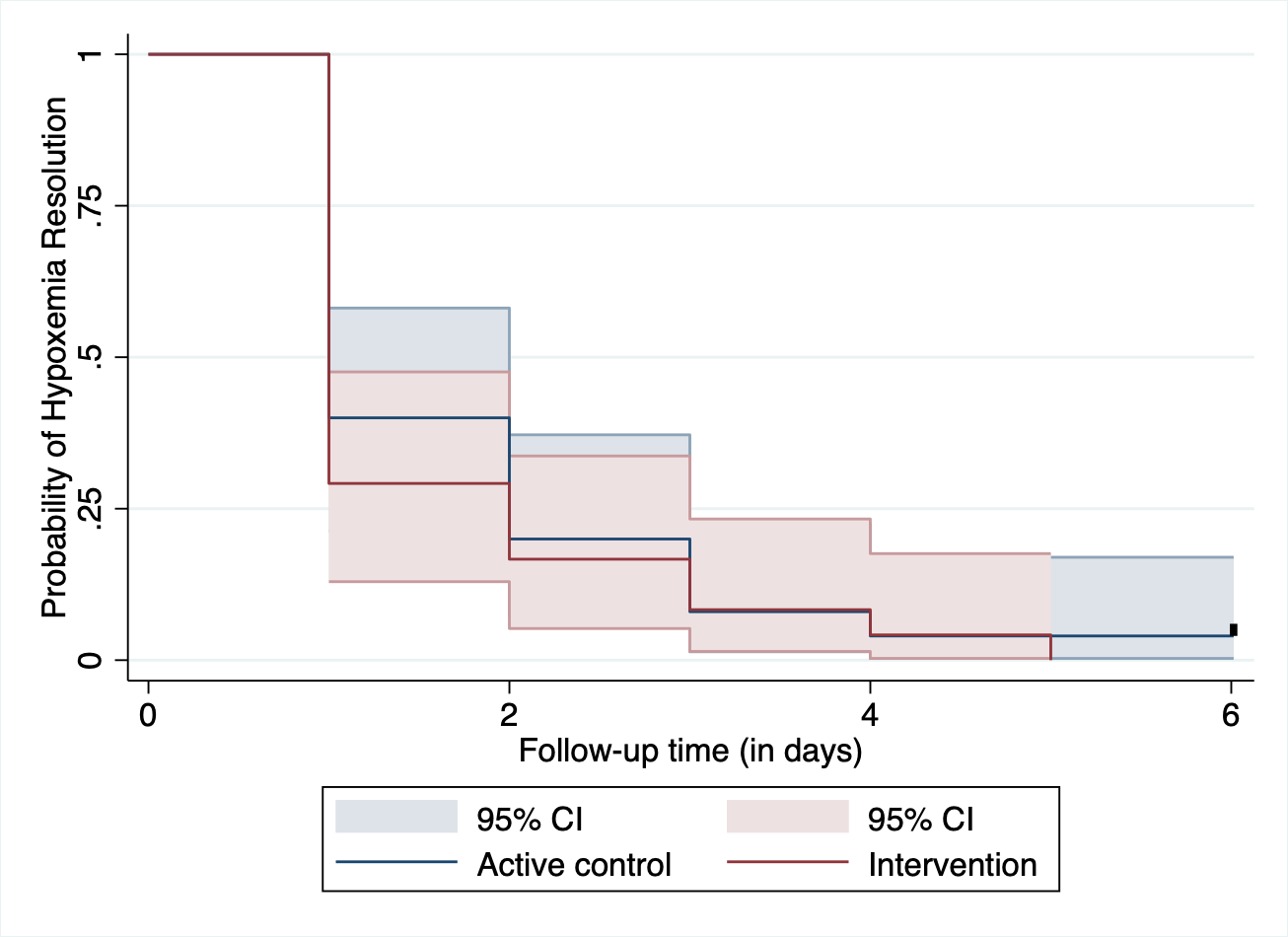


**Figure S2:**  Between group comparison of resolution of hypoxaemia

*CI, confidence interval*

p = 0.563

**Table S7**: Exploratory and follow up outcomes

| Outcome | Control | Intervention | p |
| --- | --- | --- | --- |
| Reason for ICU re/admission  Respiratory  Non-respiratory reason | 0 (0%)  1 (4%) | 0 (0%)  1 (4%) | 1 |
| Reason for reintubation  Respiratory failure  Return to theatre | 0 (0%)  2 (8%) | 0 (0%)  1 (4%) | 1 |
| Length of stay (days)  Acute hospital (postoperative)  ICU post recruitment | 6 (5-13)  29^a^ | 7 (6-14)  2 (3) | 0.42 |
| DASI /58  90 days postop | 28 (17) | 25 (12) | 0.26 |
| EQ 5D 5L – VAS /100  Postop day 5-7  90 days  EQ 5D 5L Total /25  Postop day 5-7  90 days | 56 (22)  73 (19)  10 (8-16)  7 (5-10) | 63 (21)  72 (19)  8 (7-14)  6 (6-8) | 0.22  0.91  0.08  0.58 |
| Hospital readmission incidence  30 days  90 days | 3 (1%)  7 (29%) | 6 (25%)  9 (43%) | 0.29  0.55 |
| Days alive and out of hospital  30 days  90 days | 24 (19 –26)  82 (78-86) | 20 (12 –24)  80 (71–84) | 0.11  0.15 |
| Mortality  In hospital  30 days  90 days | 1 (4%)  0 (0%)  1 (4%) | 0 (0%)  0 (0%)  0 (0%) | 1  1 |

*DASI, Duke Activity Status Index; EQ 5D 5L EuroQuol; ICU, Intensive Care Unit*

*Data are expressed as n (%), mean (standard deviation) or median (interquartile range) ^a^No standard deviation is shown as n=1 for ICU post recruitment in the control group*

**Table S8**: Between group comparison for change in Quality of life and activity status pre-admission to 90 days postop

|  | Coefficient change | 95% CI | p |
| --- | --- | --- | --- |
| EQ 5D 5L domain |  |  |  |
| Mobility | 0.07 | -0.36 to 0.51 | 0.72 |
| Personal care | -0.05 | -0.57 to 0.47 | 0.85 |
| Usual activities | -0.35 | -1.04 to 0.33 | 0.30 |
| Pain / Discomfort | -0.09 | -0.71 to 0.54 | 0.78 |
| Anxiety / Depression | -0.12 | -0.73 to 0.49 | 0.69 |
| EQ 5D 5L total | -0.67 | -2.91 to 1.57 | 0.55 |
| EQ 5D VAS | 2.0 | -11.42 to -15.45 | 0.76 |
| DASI | -3.03 | -10.34 to 4.27 | 0.41 |

*DASI, Duke Activity Status Index; EQ 5D 5L, EuroQuol; VAS, Visual analogue scale*
